# Evidence synthesis in prognosis research

**DOI:** 10.1186/s41512-019-0059-4

**Published:** 2019-07-11

**Authors:** Thomas P.A. Debray, Valentijn M.T. de Jong, Karel G.M. Moons, Richard D. Riley

**Affiliations:** 1 Julius Center for Health Sciences and Primary Care, University Medical Center Utrecht, Utrecht University, Universiteitsweg 100, Utrecht, 3584 CG The Netherlands; 2 Cochrane Netherlands, University Medical Center Utrecht, Universiteitsweg 100, Utrecht, 3584 CG The Netherlands; 3 Research Institute for Primary Care & Health Sciences, Keele University, Staffordshire, ST5 5BG UK

**Keywords:** Prediction, Meta-analysis, Prognosis, Validation, IPD

## Abstract

Over the past few years, evidence synthesis has become essential to investigate and improve the generalizability of medical research findings. This strategy often involves a meta-analysis to formally summarize quantities of interest, such as relative treatment effect estimates. The use of meta-analysis methods is, however, less straightforward in prognosis research because substantial variation exists in research objectives, analysis methods and the level of reported evidence.

We present a gentle overview of statistical methods that can be used to summarize data of prognostic factor and prognostic model studies. We discuss how aggregate data, individual participant data, or a combination thereof can be combined through meta-analysis methods. Recent examples are provided throughout to illustrate the various methods.

## Content

Thorough and systematic appraisal of the existing evidence has become mainstream in medical research and practice [1, 2]. Over the past few decades, meta-analysis has been put forward as the de facto statistical method for summarizing the results from a systematic review and appraisal of existing data on a certain topic. In meta-analysis, estimates of interest (e.g., for a specific treatment effect [3] or diagnostic test-outcome association) are obtained from individual studies and then combined into a weighted average. Such quantitative data synthesis potentially increases statistical power to detect genuine associations or effects, to investigate sources of variation within and across studies, and to answer questions that were not posed by individual studies [4, 5].

Meta-analysis is commonly applied in the domain of randomized therapeutic intervention studies [3] and, more recently, in that of diagnostic test accuracy studies. In the current era of personalized or precision medicine, the use of prognostic information is considered increasingly important to predict outcomes of individuals (off or on treatment) in order to make tailored treatment decisions [6–11]. It therefore seems timely to apply meta-analytic approaches that allow the quantitative synthesis of prognostic evidence [12].

Key barriers of quantitative synthesis of data from prognosis studies are, among others, the lack of high-quality data often due to poor reporting, lack of uniformity in statistical analysis across studies, lack of agreement on relevant statistical measures, and lack of meta-analytical guidance for synthesis of prognosis study data. Recently, much guidance has been written on how to define a review question [13], define the PICOTS (Patients, Index prognostic factor or model, Comparator factor or model, Outcomes, Timing of prognostication, Setting of prognostication), define the search strategy, design the data extraction list [14], and do risk of bias assessments [14, 15]. However, there is relatively little guidance on how to do the actual meta-analysis of results from prognosis studies.

In this paper, we discuss how the data or prognostic results from individual studies, routine care sources (e.g., hospital records or registries), and biobanks can be combined quantitatively. Hereto, we describe statistical methods for the meta-analysis of aggregate data (AD), individual participant data (IPD), or a combination thereof. The aim of this gentle overview is to inform researchers of available methods for synthesis of data of prognostic factor and prognostic model studies and to encourage their use when individual studies fail to provide generalizable evidence, as we wish to highlight recent advances in these fields.

## Quantitative synthesis in prognostic factor research

Estimates of overall prognosis (e.g., population outcome risk) are rarely sufficient to inform treatment recommendations and individual patient management. For this reason, it is often helpful to distinguish groups of people with a different average prognosis [6, 7]. A common approach is to identify specific factors that, among people with a given startpoint (such as diagnosis of disease), are associated with a subsequent endpoint [8]. This generally requires estimation of a factor-outcome association which can, for instance, be quantified using a hazard ratio or an odds ratio [8].

Several meta-analysis methods can be used to generate summary estimates of the association between a prognostic factor and a certain outcome. Although it is fairly straightforward to summarize crude (i.e., unadjusted) estimates of a particular factor-outcome association, this practice is generally discouraged because in practice hardly any prognostication is done based on a single factor only [16, 17]. For this reason, we here focus on meta-analysis methods to summarize the adjusted estimates of a certain prognostic factor and outcome. An overview of the presented methods is provided in Table 1.

**Table 1:** Available methods for quantitative synthesis in prognostic factor research

Available data		Estimate of interest	Possible methods for evidence synthesis
AD	Baseline characteristics	Linear FOA	Meta-regression [34]
	Similarly adjusted FOAs	Linear FOA	Univariate meta-analysis [19], multivariate meta-analysis [20, 33, 57]
		Non-linear FOA	Univariate meta-analysis [36, 37], multivariate meta-analysis [35]
	Not similarly adjusted FOAs	Linear FOA	Multivariate meta-analysis [33, 56, 57]
IPD		Linear FOA	One-stage meta-analysis [34, 38], two-stage meta-analysis [38], multivariate
			meta-analysis [38, 56], graphical meta-analysis [98]
		Non-linear FOA	One-stage meta-analysis [34, 41], two-stage meta-analysis [37, 41], multivariate
			meta-analysis [43]
IPD + AD	Baseline characteristiscs	Linear FOA	Hierarchical-related regression [34]
		Non-linear FOA	Hierarchical-related regression [34]
	Similarly adjusted FOAs	Linear FOA	Two-stage meta-analysis, hierarchical-related regression [49]
		Non-linear FOA	Two-stage meta-analysis [37], hierarchical-related regression [34]
	Not similarly adjusted FOAs	Linear FOA	Multivariate meta-analysis [51, 56], adaptation method [53, 54]

*FOA* factor-outcome association

### Meta-analysis of prognostic factor estimates using aggregate data

A relatively simple situation arises when the prognostic factor of interest is unadjusted in all studies, or has been adjusted for the same other prognostic factors (covariates) in all studies. Traditional meta-analysis methods—as used in meta-analysis of intervention studies—can then be used to summarize the corresponding aggregate data (AD) [18]. The most well-known approach, also from other types of meta analysis, is the so-called fixed effect meta-analysis approach, which can be formulated as follows [19, 20]:

1 $$\begin{array}{*{20}l} \hat \theta_{i} \sim \mathcal{N}\left(\mu, \hat{s}_{i}^{2} \right)  \end{array} $$

where ${\hat \theta }_{i}$ is the estimated factor-outcome association (e.g., log hazard ratio) from the *i*^th^ study, with an estimated standard error $\hat s_{i}$. This approach yields a summary estimate of the prognostic effect (*μ*), which simply represents a weighted average of the $\hat \theta _{i}$s.

A common interpretation of fixed effect meta-analysis is that the *true* factor-outcome association is identical for all studies (i.e., *θ*_*i*_=*μ*). In practice, however, true values for factor-outcome associations are likely to vary across studies due to differences in, e.g., study design, follow-up, variable definitions, adjustment factors, settings, and healthcare standards. It may therefore be more reasonable to assume that the factor-outcome associations *θ*_*i*_ are unrelated and to adopt a fixed effects meta-analysis [21]. In this approach, the weight for each study is proportional to both the number of study participants and to how much information is contributed per subject. The meta-analysis then produces an average effect applicable to an amalgamation of the contributing study populations.

Finally, a third option is to adopt a so-called random effects meta-analysis approach, which assumes that the factor-outcome associations *θ*_*i*_ are different but related across studies. A major advantage of this approach is that the presence of between-study heterogeneity can directly be quantified [19, 20]:

2 $$\begin{array}{*{20}l} \hat \theta_{i} \sim \mathcal{N}\left(\mu, \tau^{2} + \hat{s}_{i}^{2} \right)  \end{array} $$

The random effects model includes an additional parameter *τ* representing the (unknown) between-study standard deviation. The overall summary result (*μ*) now represents the average (mean) prognostic effect of the factor across the studies.

Several methods exist for estimating the weighted average *μ* and the between-study standard deviation *τ* [22, 23]. One approach is to estimate *μ* and *τ* simultaneously, e.g., by adopting (restricted) maximum likelihood estimation. Alternatively, it is possible to first estimate *τ* and then use the corresponding value to obtain an estimate for *μ*. When this strategy does not take the uncertainty of *τ* into account, confidence intervals for *μ* may become too narrow [24]. For this reason, it is generally recommended to adjust these intervals using the methods proposed by Hartung and Knapp [25] and Sidik and Jonkman [26].

**Fig. 1:**
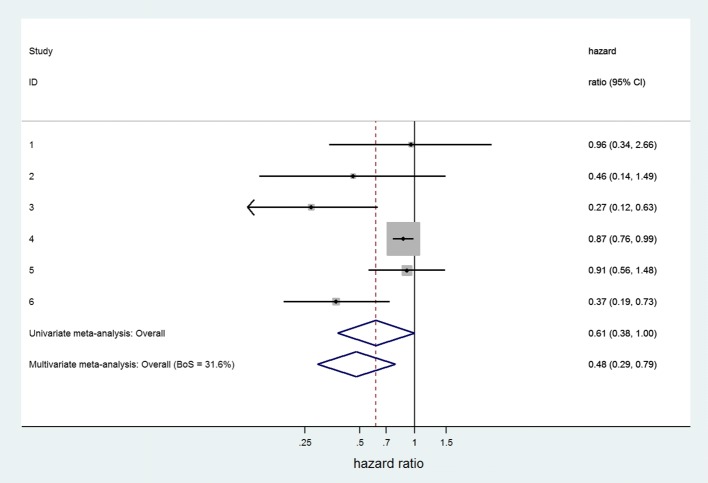
Forest plot for prognostic effect of progesterone on cancer specific survival in endometrial cancer, with summary results for univariate and multivariate meta-analysis. The multivariate meta-analysis of cancer specific survival and progression-free survival used the approach of Riley et al. to handle missing within study correlations, through restricted maximum likelihood estimation [33]. Heterogeneity was similar in both univariate and multivariate meta-analyses (*I*^2^ = 70%)

As an example, Zhang et al. previously investigated the prognostic effect of progesterone receptor status in cancer-specific survival in endometrial cancer [27]. Aggregate data from 6 studies were pooled using a random effects meta-analysis (Der Simonian and Laird method), yielding a summary hazard ratio of 0.62 and a corresponding 95% confidence interval (95% CI) ranging from 0.42 to 0.93. When adopting restricted maximum likelihood estimation, the summary estimate changed to 0.61 with a 95% CI from 0.38 to 1.00 (Fig. 1). The wider CI is due to a larger estimate of *τ* when using restricted maximum likelihood estimation rather than DerSimonian and Laird.

#### Multivariate meta-analysis

Whereas traditional meta-analysis methods are applied to summarize multiple estimates of a single parameter, it is also possible to jointly summarize multiple estimates of two (or more) parameters using so-called bivariate (or multivariate) meta-analysis methods [20, 28, 29]. These methods are well known in the meta-analysis of diagnostic test accuracy, where one jointly estimates the sensitivity and specificity of the test under review [30]. Multivariate meta-analysis methods aim to account for the correlation between the different parameter estimates and can therefore be used to deal with situations where two or more correlated parameters/statistics are to be synthesized per study. The (bivariate) random effects model for jointly summarizing the AD for two parameters of interest is given as follows:

3 $$ {}\left(\!\begin{array}{cc} {\hat {\theta}}_{1i} \\ \hat \theta_{2i} \end{array}\!\right) \!\sim\! \mathcal{N}\!\left(\!\!\left(\!\begin{array}{cc} \mu_{1} \\ \mu_{2} \end{array}\!\right)\!,\! \left(\!\begin{array}{cc} \tau_{1}^{2} & \rho \tau_{1} \tau_{2} \\ \rho \tau_{1} \tau_{2} & \tau_{2}^{2} \end{array}\!\right) \,+\, \left(\!\begin{array}{cc} \hat s_{i1}^{2} & \hat r_{i} \hat {s}_{i1} \hat s_{i2} \\ \hat r_{i} \hat s_{i1} \hat{s}_{i2} & \hat{s}_{i2}^{2} \end{array}\!\right) \!\!\right)\!  $$

where $\hat r_{i}$ and *ρ* represent the (estimated) within-study and, respectively, the (unknown) between-study correlation coefficients. For example, $\hat {\theta }_{1}$ and $\hat {\theta }_{2}$ may be the prognostic effect on outcome 1 and outcome 2, respectively.

A common application of multivariate meta-analysis arises when researchers are interested in a prognostic factor’s association with multiple outcomes [28]. For instance, in the endometrial cancer example, the unadjusted hazard ratio (HR) of progesterone was estimated for cancer-specific survival (6 studies) and for progression-free survival (11 studies). The corresponding hazard ratios of the 17 studies were then jointly pooled using a bivariate random effects meta-analysis [28]. As illustrated in Fig. 1, this strategy yielded a different and more precise summary estimate of cancer-specific survival (unadjusted HR = 0.48, 95% CI 0.29 to 0.79) as compared to the univariate meta-analysis approach above (unadjusted HR = 0.61, 95% CI 0.38 to 1.00).

Multivariate meta-analysis can also be used to jointly summarize prognostic factor-outcome associations that have been adjusted for different sets of prognostic factors (covariates). Researchers then need to distinguish between estimates that are adjusted for all relevant covariates, and estimates that are only adjusted for some (but not all) of the relevant covariates.

Unfortunately, the within-study correlations $\hat r_{i}$ are rarely reported, thereby complicating the multivariate meta-analysis approach. Riley previously demonstrated that simply ignoring these correlations can lead to meta-analysis results with inferior statistical properties [31]. Researchers may therefore assume a common within-study correlation (e.g., $\hat r_{i} = 0$ for all studies), recover its magnitude from reported summary statistics [32], or replace all within- and between-study correlations by an overall correlation parameter that is estimated from the AD at hand [33].

#### Other meta-analysis approaches

Several extensions for AD meta-analysis of prognostic factor studies have been proposed and can be used to explore sources of between-study heterogeneity [20, 34], to combine studies with different methods of measurement [35], or to combine studies that categorized continuous factors [35–37].

### Meta-analysis using individual participant data (IPD)

When IPD are available from multiple prognostic factor studies, various random effects meta-analysis models are possible that employ a one-stage or two-stage approach [3, 38, 39].

#### Two-stage meta-analysis

**Fig. 2:**
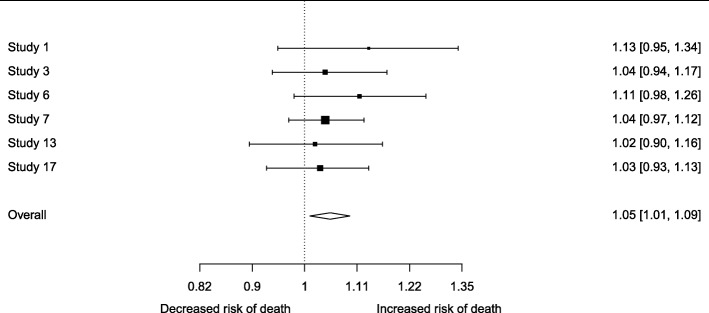
Meta-analysis of multivariable predictor effects. Association between risk of death and increase of one microvessel count, as measured by the Chalkley method. Estimates represent multivariable hazard ratios, adjusted for age and cancer stage [40]

In the two-stage approach, each study is first summarized by its factor-outcome association estimate and standard error. These AD are then appropriately combined across studies into a summary effect using traditional meta-analysis methods. For instance, Trivella et al. performed a two-stage IPD-MA to investigate the role of angiogenesis as a prognostic factor in patients with non-small-cell lung carcinoma [40]. They estimated the log hazard ratio of microvessel-density counts for each participating study center, adjusted for age and cancer stage. These estimates were then pooled using random effects inverse-variance meta-analysis (Fig. 2).

The two-stage IPD-MA approach can also be used to summarize the association of non-linear prognostic factors [41, 42]. In the first stage, the factor-outcome association of interest is modeled separately for each study with a certain functional form (e.g., cubic spline) and parameterization (e.g., location of knots). An overall function can then be obtained in the second stage by meta-analyzing the study-specific function values for distinct factor values [41, 42].

For instance, Sauerbrei et al. combined IPD from nine population-based registries to study the prognostic effect of age in T1-2 breast cancer patients [41]. They estimated a Cox regression model separately in each registry, and adjusted for 5 to 10 other prognostic factors such as the type of surgery and radiotherapy. Studywise selected fractional polynomials (FP) were used to model the adjusted effect of age. The resulting FP functions were then averaged pointwise, with weights for each registry depending on the variance of the the log relative hazard at distinct age values. Results indicated that the mortality risk is low for women between about 40 and 65 years, and increases outside this range.

#### Multivariate (two-stage) meta-analysis

Also for IPD meta-analysis, it is possible to simultaneously analyze multiple outcomes by adopting multivariate meta-analysis methods. This typically involves a two-stage approach where the IPD of each study is first reduced to AD (including estimates of the within-study correlation) and subsequently pooled across studies. Multivariate meta-analysis methods have, for instance, been proposed to summarize the association of (non-linear) continuous markers [43]. In the first stage, a common function (e.g., spline with a common location and number of knots for all studies) is estimated separately in each study. The resulting AD (e.g., multivariable regression coefficients) are then pooled across studies in the second stage. In contrast to univariate pooling of estimated effects on a grid of exposure values [41], a major advantage of this approach is that it better accounts for correlations, thereby decreasing bias and improving precision.

#### One-stage meta-analysis

An alternative approach for IPD meta-analysis (IPD-MA) of prognostic factor studies is a one-stage approach which synthesizes the IPD from all studies in a single step, while accounting for clustering of patients within studies [44, 45]. The estimation of a pooled factor-outcome association then involves the fitting of a mixed effect model, where each parameter (e.g., regression coefficient) can be specified as common, random or independent (fixed) across studies. One-stage methods appear particularly advantageous when few studies or few patients per study are available [38], or when studies involve time-to-event outcomes [46, 47].

For instance, Den Ruijter et al. performed a one-stage meta-analysis using IPD from 14 cohorts to estimate the association between (log-transformed) carotid intima-media thickness (CIMT) and the incidence of first-time myocardial infarction or stroke [48]. They first assessed between-study heterogeneity by estimating statistical interaction between cohort and CIMT measurements. Subsequently, a multivariable Cox proportional-hazards model was fitted with random effects for the baseline hazard and common effects for the regression coefficients.

When adopting a one-stage approach, it is generally recommended to account for potential ecological bias [34]. This bias may, for instance, arise when patient outcomes are associated with the mean value of the prognostic factor, rather than the individual covariate values. Ecological bias can be mitigated by separating the within-study and across-study associations, as described elsewhere [49].

### Meta-analysis using IPD and AD

Although IPD meta-analyses are generally considered as the gold standard, IPD cannot always be obtained from all relevant studies. To avoid (data availability) bias, it is often helpful to supplement the available IPD with AD for those studies where IPD are not available [50]. This strategy can be implemented using the approaches described below, assuming suitable AD can be obtained from the non-IPD studies.

#### Two-stage meta-analysis

A simple approach is to generate AD from each available IPD set and to jointly summarize the newly derived (from IPD studies) and previously published AD (from non-IPD studies) using aforementioned meta-analysis methods for AD [50]. When critical information from the non-IPD studies is missing (e.g., within-study correlations), the IPD studies can be used to derive the relevant statistics, thereby reducing the risk of bias in summary estimates [31, 35, 51, 52].

A specific situation arises when the non-IPD studies provide factor-outcome associations that are not adjusted for all relevant covariates. A two-stage bivariate meta-analysis can then be used to combine these partially adjusted estimates with the (fully and partially adjusted) factor-outcome associations from the IPD studies.

#### The adaptation method

As mentioned earlier, it is common that AD studies do not adjust for all relevant covariates and only provide factor-outcome associations that are partially adjusted. An alternative method to combine fully adjusted associations with the partially adjusted ones is to use the difference in value between the corresponding regression coefficient(s) [53, 54]. This difference is first estimated in the IPD at hand, and then applied to the summary estimate of the partially adjusted factor-outcome association. The adaptation method has, for instance, been applied in a study investigating risk factors for methicillin-resistant *Staphylococcus aureus* acute bacterial skin and skin structure infections [55]. The study authors conducted a literature review to retrieve unadjusted odds ratios for 7 potential risk factors. These odds ratios were then summarized for each risk factor using a random effects meta-analysis and adapted into an adjusted odds ratio using the IPD at hand.

The adaptation method is strongly related, and in some situations even equivalent, to the aforementioned two-stage meta-analysis approach [56]. Although formal comparisons are lacking, it has been argued that the adaptation method may be less statistically and computationally efficient.

#### Hierarchical-related regression

This one-stage approach directly combines the available IPD and AD by specifying a distinct likelihood for each data source [34, 49]. This enables the IPD studies to contribute in all parameter estimates, whereas the AD studies are only used to estimate the study-level parameters and across-study relationships. For example, Riley and Steyerberg adopted hierarchical-related regression to investigate the relationship between age and the risk of 6-month mortality in patients with traumatic brain injury (TBI) [34]. They used a Bernoulli distribution to model the binary outcomes from 4 IPD studies and a Binomial distribution for the observed event counts in 10 AD studies. To account for potential ecological bias, the within-study and across-study effects for participant age were separated when jointly analyzing the 14 studies. It was found that an individual’s probability of death by 6 months increases as their individual age increases and also as the mean age in their study (or population) increases. A possible explanation for this is that studies with a higher mean age involved clinicians with less experience of treating TBI patients.

### Summary points

Evidence synthesis in prognostic factor research may help to identify factors that are associated with a certain clinical outcome, to explore their functional form, and to quantify their incremental value over established prognostic factors [8]. When IPD are unavailable, traditional meta-analysis methods can be used to summarize published prognostic factor estimates in order to identify genuine prognostic factors [18]. Although IPD are not strictly required to assess the incremental value of a prognostic factor or to explore its functional form, this may often be unfeasible using published AD only [44]. For this reason, when IPD are available for a few studies, corresponding information can be used to restore unreported AD (e.g., missing within-study correlations) or to adapt unadjusted factor-outcome associations. Evidence synthesis in prognostic factor research is, however, most appealing when multiple sources of IPD are available, as this allows to derive desired prognostic factor results directly and to analyze continuous factors more appropriately [8]. Meta-analysis of IPD is preferably initiated using a two-stage approach, as corresponding methods are relatively straightforward to implement and guard against ecological bias. One-stage meta-analysis methods may, however, be more appealing when few studies or few subjects per study are available, as they are more flexible, resistant against small sample bias, and avoid the need for estimating correlations between random effects [38].

## Quantitative synthesis in prognostic model research

Prognostic model research aims to examine multiple prognostic factors in combination [6], in order to predict the absolute risk of future outcomes in single individuals. Corresponding studies may derive new prognostic models (so-called development studies), evaluate the performance of existing models in new individuals (so-called validation studies) and if necessary tailor their predictions, or examine the model’s impact on health-related outcomes.

**Fig. 3:**
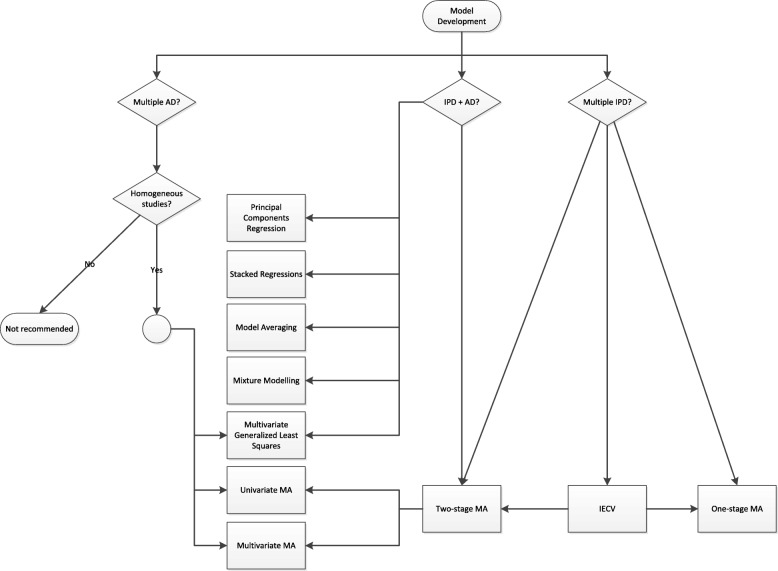
Available methods for quantitative synthesis during prognostic model development. Abbreviations: MA, meta-analysis; IECV, internal-external cross-validation; AD, aggregate data; IPD, individual participant data

**Fig. 4:**
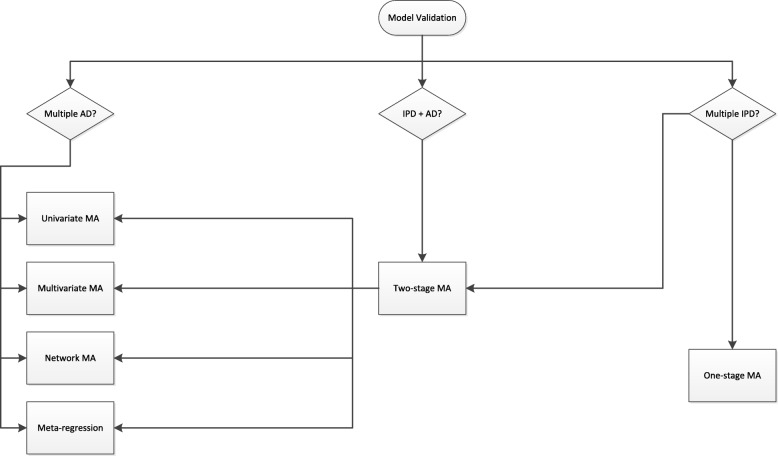
Available methods for quantitative synthesis during prognostic model validation. Abbreviations: MA, meta-analysis; AD, aggregate data; IPD, individual participant data

Currently, most prognostic models are developed based on relatively small studies. Hence, many of these models do not perform adequately when applied to other individuals [9, 58–60]. To investigate and improve the performance of prognostic models across different settings and populations, researchers may consider meta-analysis methods during their development and validation [6, 61–65]. Several strategies for this purpose are described below and summarized in Figs. 3 and 4. As before, we distinguish between situations where the available data sources comprise of aggregate data, individual participant data, or a combination of both.

### Meta-analysis using AD

#### Validation of an existing prognostic model

A common source of AD are so-called external validation studies assessing the (discrimination and calibration) performance of a certain prognostic model when tested in other individuals than from which the model was developed. By summarizing these performance estimates, it becomes possible to identify whether the model’s predictions are sufficiently accurate across different settings and populations. This typically requires the retrieval of multiple performance statistics (e.g., concordance statistic, calibration-in-the-large, calibration slope) and corresponding standard errors [66, 67]. The resulting estimates can then be pooled using traditional meta-analysis methods, provided that an appropriate scale [68] or link function [67, 69] is used. Although different study weights can be used [21, 70], it is generally recommended to allow for between-study heterogeneity as validation studies are likely to differ in their design and execution [66–68]. As is the case in meta-analysis of prognostic factor research, meta-regression can be used to explore potential sources of between-study heterogeneity.

For instance, van Doorn et al. reviewed 19 published validations of CHA2DS2-VASc, a prediction model for estimating stroke risk in patients with atrial fibrillation [71]. A random effects meta-analysis was applied to summarize estimates of model discrimination (logit c-statistic) and annual risk per score (square root risks). The summary c-statistic was 0.64 (95% CI 0.56–0.71), which increased to 0.71 (95% CI 0.62–0.79) for studies recruiting patients from a hospital care setting. Further, stroke risks were found to vary substantially within the different scores and were notably elevated in hospital patients as compared to patients from the general population.

#### Development of a new prognostic model

It is also possible to summarize AD from multiple but similar prognostic model development studies and to combine their regression coefficients into a new prediction model (for example, via a multivariate meta-analysis) [32, 57]. This strategy is, however, often complicated by the poor reporting of key model parameters (and their standard errors and within-study correlations), by inconsistent covariate adjustment across studies, and by the presence of between-study heterogeneity. For this reason, meta-analysis of previously developed prognostic models only seems reasonable when the corresponding studies are fairly homogeneous and when the required AD are reported in sufficient detail (see also Fig. 3).

### Meta-analysis using IPD

When IPD are available, it becomes possible to assess and optimize the prognostic model’s performance across different settings and populations using a one-stage or a two-stage meta-analysis approach.

#### Validation of an existing prognostic model

In the two-stage approach, the model is first validated separately in each IPD, yielding study-specific estimates of model discrimination and calibration. These estimates are then pooled across studies in the second stage, using univariate [66, 70, 72] or multivariate [73] meta-analysis methods (Fig. 4). For instance, Snell et al. adopted multivariate IPD meta-analysis to summarize the calibration slope and concordance statistic of a prognostic model for breast cancer incidence. The summary estimates were then used in combination with estimates of between-study heterogeneity to calculate the probability that model performance would be adequate (i.e., within certain ranges) in new populations [73].

Model validation can also be performed through a one-stage approach. For instance, the summary calibration slope can be derived by fitting a mixed effect model with study-specific intercept terms and a random effect for the prognostic index.

**Fig. 5:**
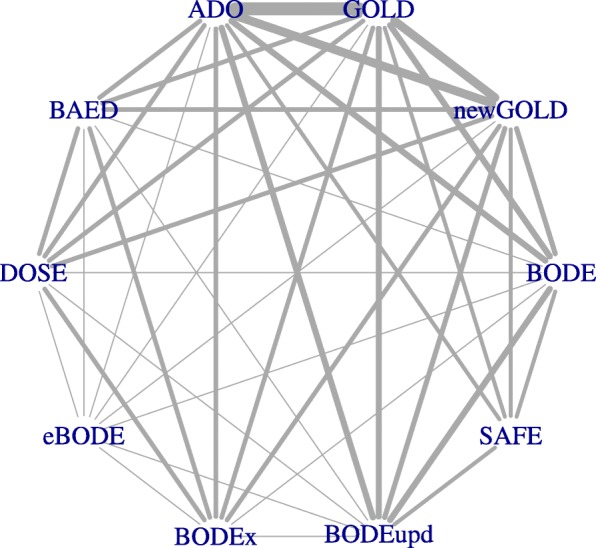
Validation of 10 prognostic models for 3-year mortality in patients with chronic obstructive pulmonary disease.Depiction of network structure with lines weighted by the total number of participants available for each model comparison [74]. Abbreviations: GOLD, Global initiative for chronic Obstructive Lung Disease; BODE, Body mass index, airflow Obstruction, Dyspnoea and severe Exacerbations; BODE upd., BODE updated; ADO, Age, Dyspnoea, airflow Obstruction (we use the updated version of the ADO score in our analysis); e-BODE, severe acute exacerbation of COPD plus BODE; BODEx, Body mass index, airflow Obstruction, Dyspnoea, severe acute Exacerbation of COPD; DOSE, Dyspnoea, Obstruction, Smoking and Exacerbation frequency; SAFE, Saint George’s Respiratory Questionnaire (SGRQ) score, Air-Flow limitation and Exercise capacity; B-AE-D, Body-mass index, Acute Exacerbations, Dyspnoea

Finally, several extensions of one-stage and two-stage meta-analysis are possible. For instance, network meta-analysis (NMA) can be used to assess the (relative) performance of multiple prognostic models [74], which is particularly helpful when direct comparisons are not feasible for some studies. As an example, Haile et al. compared the performance of 10 prognostic models for calculating mortality risk in patients with chronic obstructive pulmonary disease [74]. Although IPD were available for 24 cohort studies (*N*=15 762), information on important variables was often missing such that some models could not be validated in one or more studies (Fig. 5). A two-stage NMA was therefore adopted to summarize all available evidence on the models’ comparative performance and to allow the inclusion of studies where only few models could be validated.

#### Development of a new prognostic model

Meta-analysis of IPD is used increasingly often to develop new prognostic models, with improved generalizability across different settings and populations. Meta-analysis approaches are similar to prognostic factor research, and may involve a one-stage or a two-stage approach (see also Fig. 3) [70]. In the two-stage approach, the parameters of the prognostic model (e.g. intercept term and regression coefficients) are estimated separately in each study and subsequently combined across studies using either a fixed or random effects meta-analysis. Conversely, in the one-stage approach, all IPD are simultaneously analyzed by assuming a common, fixed, or random effect for each model parameter. Both approaches then yield a set of study-specific and/or “pooled” regression coefficients that can be used for making absolute risk predictions in a variety of populations. One-stage approaches are particularly helpful when studies are relatively small, or contain few events, as they use a more exact statistical approach and do not require continuity corrections when (partial) separation occurs [38]. Conversely, two-stage approaches are generally preferred when modeling interactions or non-linear terms, as they guard against over-parameterization and ecological bias [43].

As an example, Westeneng et al. recently performed a meta-analysis with IPD from 14 European cohorts to develop the ESCALC model for predicting survival in patients with amyotrophic lateral sclerosis [75]. They fitted a Royston-Parmar survival model in the entire set of *N*=11 475 patients and assumed a common baseline hazard and regression coefficients across cohorts. Because the resulting model showed some extent of mis-calibration upon validation, recalibrated cohort-specific baseline hazard functions were reported to enable researchers to tailor model predictions to their population.

A particular advantage of IPD meta-analysis is that it enables the direct evaluation and optimization of a model’s generalizability across different settings and populations through internal-external cross-validation [64, 65, 76–78]. Briefly, this method iteratively omits one study from the meta-analysis to externally validate a model that is developed on the remaining studies. This process is repeated several times, leading to multiple estimates of model performance, which in turn can be summarized using aforementioned meta-analysis methods [68, 73]. If performance appears adequate across the available studies, the pooled data is used to develop a final model. Otherwise, it flags heterogeneous study populations where a developed model might not perform well and signals that additional predictors or more advanced modeling approaches (such as the inclusion of non-linear terms) or updating strategies (such as recalibration) might be needed.

Internal-external cross-validation has, for instance, been adopted during the development of ESCALC, a prognostic model for predicting survival in patients with amyotrophic lateral sclerosis. A one-stage approach was used to estimate a Royston-Parmar model using IPD from all but one study, after which its external validity was evaluated in the omitted study. The process was repeated for all studies, providing 14 estimates of discrimination and calibration performance. These estimates were then pooled using a random effects meta-analysis, yielding a summary c-statistic and calibration slope of, respectively, 0.78 (95% PI 0.74 to 0.82) and 1.01 (95% PI 0.83 to 1.18). These results suggest that the model is likely to perform well across different settings and populations.

### Meta-analysis using IPD and AD

#### Validation of an existing prognostic model

Because IPD is commonly unavailable for one or more relevant validation studies, researchers may consider a two-stage meta-analysis to combine published estimates of prediction model performance with those derived from the IPD at hand. This approach has, however, not extensively been studied yet, and further research is also warranted to explore alternative strategies such as hierarchical-related regression.

#### Development of a new prognostic model

For many disease areas, there is an abundance of competing models that predict similar outcomes in related populations. Hence, rather than developing a new prognostic model from scratch, it can be advantageous to combine the AD of the existing models with the available IPD [79–82]. One approach is to summarize the models’ regression coefficients together with the associations from the IPD [51, 52]. This is particularly useful if the data are reasonably homogeneous, as synthesis then yields a prognostic model that is applicable to the “average” population. Conversely, when studies have different baseline risk or predictor-outcome associations, some tailoring will often be necessary to ensure that the new model remains sufficiently accurate in local settings. In these situations, the IPD can be used to adjust the existing models to specific populations by adopting Bayesian inference [52], model averaging [81], regression analysis [79, 81, 83, 84], or mixture models [83].

For example, model averaging was recently applied to combine the logistic EuroSCORE and EuroSCORE II models for predicting short-term mortality in patients undergoing coronary artery bypass graft surgery [82]. These models showed substantial mis-calibration in contemporary registry data and were therefore combined into a single model that was tailored to the contemporary population.

### Summary points

Many prognostic model studies are based on relatively small samples, leading to overfitting, poor generalizability, and over-optimism [58, 85]. Evidence synthesis allows to increase the effective sample size and to study more diverse settings and populations [62, 64]. Although synthesis is ideally based on IPD, a systematic review and meta-analysis of published data can initially be performed to study the (discrimination and calibration) performance of a previously developed model. Estimates of between-study heterogeneity can then help to reveal the extent of necessary improvements (e.g., local tailoring) and to calculate the probability that the model(s) will be clinically useful in certain settings [73, 86]. In general, a good model will have satisfactory performance across different settings and populations. However, if prediction model performance is poor overall or prone to substantial between-study heterogeneity, retrieval of IPD may help to study causes of detrimental performance [66, 67, 87] and to establish whether distinct models are needed for different settings and populations [61].

When developing new or updating existing models, it is important to consider heterogeneity in baseline risk, predictor effects, the linear predictor, and the absolute risk predictions [61]. Risk predictions should be reasonably similar across studies for a prediction model to be labeled “generalizable,” and therefore, it is helpful to limit any heterogeneity in baseline risk and predictor effects while keeping the model’s overall performance sufficiently high. Although internal-external cross-validation using IPD from multiple studies may be helpful to achieve this, further research is needed to integrate this endeavor in a statistical framework.

Finally, for newly developed prediction models from IPD-MA, it is helpful to provide any information that allows for tailored predictions. For instance, appropriate intercept terms can often be derived from the outcome incidence, particularly if predictor variables have been centered around their local means [77]. Similarly, predictor effects can sometimes be tailored using information about their particular measurement [88]. When it remains unclear which parameter values (e.g., intercept term) are most appropriate for predictions in new populations, researchers may use the pooled estimates or, preferably, integrate over the distribution of the random effects [89].

## Concluding remarks

In this paper, we have summarized and sign-posted various methods for meta-analysis of prognostic factor and prognostic model studies. Because these primary prognosis studies may address very different types of research questions and are often poorly reported, advanced meta-analysis methods are usually needed to provide (meaningful) summary estimates and understand sources of between-study heterogeneity. Regardless, researchers should not be daunted by their complexity, as we have shown that many of these methods have been implemented in traditional software packages and lead to an improved understanding of prognosis-related research questions.

For researchers embarking on a meta-analysis, the following issues should be taken into account. First, it is important to ensure that available data are of sufficient relevance and quality. It is recommended to conduct a systematic review of the literature and to harmonize available IPD sets. Similarity of datasets can, for instance, be improved by standardizing related measurement scales [90], by adopting measurement error correction methods [91–93], or by treating bias arising from measurement error as a missing data problem [90, 92, 94]. Second, when datasets are affected by missing data, advanced imputation methods are needed to ensure valid inferences [95–97]. Finally, it is important to realize that not all meta-analysis methods have yet been rigorously assessed and that further research is still needed to explore their potential areas of application.

## Abbreviations

AD: Aggregate data
CI: Confidence interval
CIMT: Carotid intimia-media thickness
FP: Fractional polynomial(s)
HR: Hazard ratio
IPD: Individual participant data
IPD-MA: Individual participant data meta-analysis
NMA: Network meta-analysis
TBI: Traumatic brain injury
